# Hepatic steatosis in HCV-infected persons in the direct-acting antiviral era

**DOI:** 10.1186/s40794-016-0038-5

**Published:** 2016-09-27

**Authors:** Heather L. Stevenson, Netanya S. Utay

**Affiliations:** 1 Department of Pathology, University of Texas Medical Branch, 301 University Blvd, Galveston, TX 77555 USA; 2 Division of Infectious Diseases, Department of Medicine, University of Texas Medical Branch, 301 University Blvd, Galveston, TX 77555 USA

**Keywords:** HCV, HIV, Hepatic steatosis, NAFLD, Metabolic syndrome, Cirrhosis, Direct-acting antivirals

## Abstract

Hepatitis C virus (HCV) infects 130–170 million people worldwide. Recently, direct-acting antivirals have been shown to eradicate HCV infection in 90–95 % of non-cirrhotic patients depending on genotype, treatment experience, and regimen used. Similar rates are achieved among compensated cirrhotics, although longer treatment duration and/or ribavirin may be required. HCV uses host lipid metabolism for its lifecycle and can cause hepatic steatosis and insulin resistance. Hepatic steatosis, defined as excessive triglyceride deposition in hepatocytes, affects approximately half of HCV-infected individuals. Genetic factors and co-morbidities can drive further steatosis, which in turn can instigate fibrosis and progression to cirrhosis and hepatocellular carcinoma. Polymorphisms in genes that modulate lipid deposition in hepatocytes such as patatin-like phospholipase domain-containing protein 3 (PNPLA3) and transmembrane six superfamily member 2 (TM6SF2) predispose people to steatosis. Metabolic syndrome, obesity, and insulin resistance are increasing worldwide and further contribute to hepatic steatosis, and alcohol has long been recognized as a cause of lipid deposition in the liver. HIV and antiretroviral drugs, but not HBV, may further drive hepatic steatosis. While many of these factors limit response to interferon-based regimens for treating HCV, responses to direct-acting antivirals appear not to be impaired. The effect of HCV eradication on hepatic steatosis and progression to fibrosis, cirrhosis, and hepatocellular carcinoma warrants further study in the era of direct-acting antivirals.

## Background

Hepatitis C virus (HCV) infects 130–170 million people worldwide, close to 3 % of the world’s population [1]. Approximately 80 % develop chronic viral hepatitis, which can progress to liver fibrosis, cirrhosis, and hepatocellular carcinoma (HCC). Recently, interferon (IFN)-free direct-acting antiviral regimens have been developed for HCV treatment. Despite their cost, these direct-acting antiviral regimens are now the treatment of choice for all HCV genotypes. Sustained virologic response (SVR) at 12 weeks (SVR12), i.e., undetectable HCV RNA levels 12 weeks after completing treatment, is achieved in 90–95 % of non-cirrhotics, depending on genotype, treatment experience, and regimen used [2–4]. Comparable responses can be achieved in cirrhotics, but an extended treatment duration and/or ribavirin may be required based on the regimen [2]. However, SVR12 may be achieved in only 80–85 % of decompensated cirrhotics with most regimens, although newer options approach SVR rates of 95 % [2, 4–7].

HCV-infected persons with superimposed conditions such as human immunodeficiency virus (HIV) infection or hepatic steatosis progress to fibrosis and cirrhosis more often and more quickly [8, 9]. In the IFN era, many of these co-morbidities compromised treatment success. Now, with HCV eradication possible in virtually everyone, the sequelae of steatosis and its drivers will garner more attention. Here, we explore the drivers of hepatic steatosis in persons infected with HCV and how these factors may contribute to clinical outcomes.

### HCV and steatosis

Approximately 40–80 % of HCV-positive patients that are biopsied have steatosis, defined as excessive triglyceride deposition in hepatocytes [10, 11]. The prevalence of hepatic steatosis has a strong genotype dependence, [12, 13] and genotype 3 infection is now widely accepted as an independent cause of steatosis. Increased severity of hepatic steatosis correlates with higher viral loads, and genotype 3-associated steatosis resolves after SVR with antiviral treatment [12, 14]. Although HCV genotype 3 infection is independently associated with accelerated fibrosis progression [15] and increased risk for HCC, the steatosis associated with genotype 3 has not been shown to increase these complications.

While genotype 3 patients have increased fibrosis progression, enhanced risk of HCC development, and lower SVR rates with direct-acting antivirals, genotype 1 patients with steatosis may also be at risk for poorer clinical outcomes. This steatosis is likely not mediated solely by HCV. Thus, SVR may not rectify the accelerated hepatic injury and increased fibrosis progression, although some recent studies have shown improvement in lipid profiles following successful HCV treatment in genotype 1 patients [16].

Hepatic steatosis in HCV-infected persons can also be caused by the same host factors that contribute to steatosis in HCV-uninfected persons, such as metabolic syndrome, increased body mass index (BMI), hypertriglyceridemia, chronic alcohol use, other infections, or exposure to certain medications [17]. Unlike steatosis caused by genotype 3, steatosis caused by metabolic syndrome or insulin resistance is associated with accelerated fibrosis progression, decreased response to IFN-based treatment regimens, and increased risk for HCC [15].

In many people both viral- and host-mediated factors likely contribute to the development of hepatic steatosis. Indeed, HCV replication relies on host lipid metabolism for its lifecycle and results in hepatic steatosis by several mechanisms such as enhancing lipogenesis, impairing mitochondrial lipid oxidation, and downregulating microsomal triglyceride transfer protein (MTTP) activity [18–20]. Thus, steatosis itself may enhance HCV replication, which has been shown to occur in alcohol-altered lipid metabolism [21].

### Metabolic syndrome

Obesity results in increased accumulation of fat, primarily triglycerides, which are synthesized from glycerol and long chain fatty acids (LCFA). LCFA enter hepatocytes via specific, facilitated transport processes, which are regulated in obesity at least in part by insulin, leptin, and spexin [22]. The metabolism of the increased cellular triglyceride content may lead to cell-specific lipotoxicity, contributing to several comorbidities, including hepatic steatosis, non-alcoholic fatty liver disease (NAFLD), and non-alcoholic steatohepatitis (NASH). Overall, in obesity the development of hepatic steatosis is due to too many triglycerides entering the liver via increased LCFA uptake and synthesis, increased triglyceride uptake and synthesis, and too little being removed due to decreased ApoB100 synthesis, decreased triglyceride mobilization, and decreased VLDL assembly and secretion [22].

The most frequent causes of hepatic steatosis in persons that are infected with genotypes other than type 3, include increased BMI and visceral obesity [23–25]. These two factors provide strong evidence that insulin resistance is the primary pathologic mechanism that leads to abnormal lipid accumulation within hepatocytes. However, it is difficult to determine if the insulin resistance is entirely host-driven, due to HCV infection, or a combination [20, 26–28]. With the increasing incidence of NAFLD and non-alcoholic steatohepatitis (NASH) in the HCV-infected population, underlying host factors are commonly superimposed on chronic HCV infection. HCV itself has been shown to increase insulin resistance, and insulin resistance increases with increasing viral load and decreases after HCV treatment [29–31]. Based on in vitro experiments, HCV core protein may increase insulin resistance by down-regulation of glucose transporter 2 (GLUT2), which is responsible for transportation of glucose to hepatocytes. TNF-alpha pathways have also been shown to be involved in insulin receptor substrate inhibition, leading to possible GLUT4 inhibition and decreased uptake of glucose from hepatocytes and other cells [32–35]. Just as for insulin resistance, it is difficult to determine the extent to which host factors are responsible for the development of diabetes mellitus in HCV-infected individuals versus the amount the viral infection itself contributes. Regardless, there is now convincing evidence that HCV infection increases the risk of developing diabetes, which then increases the risk of developing hepatic steatosis [20].

### Genetic predisposition to steatosis in HCV infection

Several genes have recently been identified as predisposing factors for hepatic steatosis, including in the setting of HCV infection. Patatin-like phospholipase domain-containing protein 3 (PNPLA3), a lipase that may mediate lipid deposition in hepatocytes and adipocytes [36, 37], is perhaps the best described of these factors. Increased PNPLA3 expression is associated with steatosis, with higher levels correlating with greater severity [38]. The rs738409 (C- > G) I148M polymorphism is associated with increased risk of NAFLD and progression of NAFLD to NASH and cirrhosis in HCV-uninfected persons [39–41]. This polymorphism is also associated with steatosis, steatohepatitis and fibrosis in HCV-infected persons [42–44], even after adjusting for age, sex, body mass index and diabetes [45]. Transmembrane six superfamily member 2 (TM6SF2) likewise modulates triglyceride deposition in hepatocytes. The rs58542926 (A- > G) E167K polymorphism in TM6SF2 is associated with increased steatosis in persons with chronic HCV infection [46], albeit to a lesser extent than the PNPLA3 polymorphism [47], and increased hepatic steatosis, steatohepatitis, and fibrosis in NAFLD [46, 48]. Increased hepatic steatosis and fibrosis on biopsy of HIV/HCV co-infected patients were also associated with polymorphisms in the Fat Mass and Obesity-Associated Protein (FTO) gene, which may influence food consumption [49]. Among the Chinese Han population, ApoC3 polymorphisms correlated with higher hepatic and circulating triglyceride levels and subsequently increased risk of NAFLD [50], and among diabetic Taiwanese participants, a polymorphism of the adiponectin gene rendering lower levels was associated with increased risk of NAFLD [51]. In contrast, polymorphisms resulting in increased concentrations of uncoupling protein 2 (UCP2), which modulates reactive oxygen species production, confers a decreased risk of NASH [52]. In sum, numerous genetic factors may predispose people with and without HCV infection to hepatic steatosis.

### HCV and alcohol

The development of hepatic steatosis due to chronic alcohol exposure involves several complex metabolic pathways including the alcohol dehydrogenase (ADH) pathway, microsomal ethanol-oxidizing system (MEOS), peroxisome proliferator-activated receptor-α (PPAR-α) and PPAR-γ, AMP activated protein kinase (AMPK), sterol regulatory element-binding proteins (SREBPs), endoplasmic reticulum stress and methionine metabolism, and mitochondrial abnormalities and lipid peroxidation [53]. Both acute and chronic alcohol use result in increased production of reactive oxygen species (ROS) and reductions in the levels of antioxidants, which is partly mediated by the intermediate product of ethanol metabolism, aldehyde. Due to increased activity of these various metabolic pathways, oxygen requirement by hepatocytes is increased, leading to ischemia and centrilobular liver necrosis. In addition, steatotic hepatocytes are more susceptible to ischemic injury [54].

Cytokines produced by adipocytes and inflammatory cells including Kupffer cells, such as adiponectin, leptin, TNF-α, and IL-6, also play a role in the pathogenesis of alcohol-mediated liver disease and NASH [53]. Through many interactions, including with PPAR-α and PPAR-γ, adiponectins decrease lipid synthesis and increase lipolysis. Whether alcohol results in decreased adiponectin levels remains controversial [55, 56]. Leptin increases CD14 expression on Kupffer cells and activates hepatic stellate cells, promoting hepatic inflammation and fibrosis, respectively [57]. Alcohol also increases the permeability of the gut barrier, leading to increased endotoxemia, and changes the intestinal microbiome [58, 59], which further enhances cytokine production and activation of hepatic inflammatory cells and stellate cells.

There is an increased prevalence of HCV infection in alcoholics [60, 61] and liver injury appears to be more severe in HCV-infected persons with steatohepatitis. People infected with HCV that are heavy drinkers have accelerated fibrosis progression and are at higher risk of developing HCC [62, 63]. Several factors have been implicated in potentiating the effects of alcohol exposure in HCV-infected persons, which includes impaired antiviral immunity, increased viral replication, increased oxidative stress, increased iron overload, and steatosis.

### Contribution of chronic HBV and HIV co-infections to steatosis

Approximately 2.75 million people worldwide have HIV-HCV co-infection and 2.6 million have HBV-HCV co-infection [64]. HIV infection accelerates the progression of HCV to cirrhosis [65], but HIV's contribution to steatosis has only been recognized recently. NAFLD affects 13-50 % of HIV-infected persons [66–68]. In a study of 30 HIV-infected participants with unexplained elevated transaminases, 72 % had steatosis and/or fibrosis, 53 % had NASH, and 63 % had fibrosis [69]. HIV-HCV co-infected participants have a higher rate of steatosis than either mono-infected group in some studies [70–73]. Potential contributing factors may include dyslipidemia and insulin resistance due to HIV and/or antiretrovirals, obesity, sedentary lifestyle, and increased systemic inflammation [9, 74, 75]. Older antiretrovirals such as stavudine appear more likely to contribute to steatosis than the more commonly used agents today [75]. In addition, decreased adiponectin with HIV infection may contribute to the increased steatosis [76]. Thus, lipid deposition in the liver is increased in HIV infection.

HIV-infected people with NAFLD are also more likely to develop steatohepatitis [77]. HIV can directly infect Kupffer cells, although these data are controversial [78]. Regardless, HIV may increase the population, proliferation, and turnover of Kupffer cells in the liver, resulting in a pro-inflammatory state [78]. HIV infection is associated with increased translocation of microbial products from a permeable intestinal barrier [79] through the portal circulation to the liver, where they are phagocytosed by Kupffer cells and may drive further hepatic inflammation [80]. The mitochondrial dysfunction induced by HIV and antiretrovirals induces reactive oxygen species production, causing additional hepatic damage [81]. The overall suppression of regulatory T cell pathways by HIV infection may also inhibit modulation of this inflammatory state [9]. Ultimately, HIV accelerates progression to cirrhosis in HCV-infected persons, particularly in the presence of steatosis [65, 82].

HBV increases the rate of progression to cirrhosis and hepatocellular carcinoma in HCV-infected persons [83], but whether HBV can contribute to hepatic steatosis remains controversial. The HBV protein HBx, which facilitates HBV replication [84], induces hepatic steatosis and fatty acid oxidation in mouse models [85, 86]. However, based on a meta-analysis of human studies, hepatic steatosis is less common among HBV-infected compared to HCV-infected persons [72] and is usually found in conjunction with host factors such as hypertriglyceridemia [72, 87]. Some data suggest that HBV infection may even be associated with a lower risk of steatosis, but this was also in the context of lower triglyceride levels and less metabolic syndrome [88]. Unexpectedly, increased hepatic steatosis is actually associated with lower HBV DNA levels [72, 89], but this finding may be explained by the association of PNPLA3 polymorphisms with both steatosis and lower HBV DNA levels [90]. Thus, HBV does not clearly increase the risk of steatosis.

### The effect of comorbidities on HCV treatment response

The direct-acting antiviral agents for HCV infection achieve SVR rates exceeding 95 % for non-cirrhotic patients. Lengthening duration of therapy and/or adding ribavirin can render similar SVR rates in cirrhotics, but with most regimens, SVR rates are about 10 % lower in decompensated cirrhotics [2]. Co-morbidities and concomitant medications may limit regimen options, which could influence SVR rates. Co-morbidities that contribute to cirrhosis by increasing the risk of steatohepatitis may also impact HCV treatment success.

These contributors to steatohepatitis have varying impacts on response rates to HCV treatment. Whether steatosis alone affects SVR rates to IFN-based regimens in the absence of fibrosis is controversial, and few studies have been done with IFN-free regimens [82, 91–94]. Metabolic syndrome was associated with decreased SVR rates in the IFN era [95, 96] but no longer seems to have an effect [97, 98]. People with HIV infection had lower SVR rates to IFN-based regimens than HIV-uninfected people, 40 % versus 56 % [99], but now HIV-infected persons achieve comparable SVR rates, exceeding 95 % in the absence of cirrhosis [100–102]. HBV does not decrease SVR rates regardless of the treatment regimen. However, HBV and HCV inhibit each other's replication, and consequently, HBV may reactivate with HCV treatment [103–106], with the risk for progression to cirrhosis and HCC. No data demonstrate decreased SVR rates to direct-acting antivirals with alcohol or even illicit substance use [2]. However, binge drinking, illicit substance use and potential complications such as incarceration can interfere with a patient's adherence to antiviral regimens and therefore warrant consideration. Thus, most comorbidities do not appear to directly attenuate responses to direct-acting antiviral treatment, but their contributions to cirrhosis may lower the likelihood of achieving SVR.

## Conclusions

HCV infection is now a curable disease. However, HCV-infected people have comorbidities that cause steatosis and may continue to damage the liver after HCV eradication, including the increasingly prevalent metabolic syndrome. HCV may contribute to hepatic steatosis and to metabolic syndrome, forming a positive feedback loop that may further increase steatosis and culminate in steatohepatitis and fibrosis. Ultimately, after SVR, fibrosis can regress in some patients [107, 108], but based on data from the IFN era, the presence of these comorbidities may prevent fibrosis regression. Long-term studies in HCV-infected persons treated with direct-acting antivirals will illuminate the degree to which steatosis, steatohepatitis, and/or fibrosis reverse with SVR, particularly with persistence of other comorbidities. In sum, comorbidities may have less of an impact now on SVR with the highly efficacious direct-acting antiviral therapy, but their persistence may prevent complete return to health in HCV-cured patients.

## Abbreviations

ADH: Alcohol dehydrogenase
AMPK: AMP-activated protein kinase
BMI: Body mass index
GLUT: Glucose transporter
HBV: Hepatitis B virus
HCC: Hepatocellular carcinoma
HCV: Hepatitis C virus
HIV: Human immunodeficiency virus
IFN: Interferon
LCFA: Long chain fatty acids
MEOS: Microsomal ethanol oxidizing system
MTTP: Microsomal triglyceride transfer protein
NAFLD: Non-alcoholic fatty liver disease
NASH: Non-alcoholic steatohepatitis
PNPLA3: Patatin-like phospholipase domain-containing protein
PPAR: Peroxisome proliferator-activated receptor
SREBPs: Sterol regulatory element-binding proteins
SVR: Sustained virologic response
TM6SF2: Transmembrane six superfamily member 2
UCP2: Uncoupling protein 2
